# Supplementary material for: Spanish version of Multidimensional Mentalizing Questionnaire (MMQ): Translation, adaptation and psychometric properties in a Chilean population
Source: PLoS One. 2024 Mar 18;19(3):e0296691. doi: 10.1371/journal.pone.0296691 (PMC10947660; doi:10.1371/journal.pone.0296691)
Supplement: Attachment — Submitted filename: ResponsetoReviewers.docx [file pone.0296691.s001.docx]

Response to reviewer

We thank our reviewer for his suggestions. We have addressed them all in the manuscript. With these changes and additions, our manuscript has substantially improved.

*Reviewer #1: Thank you for the invitation to review “Spanish version of Multidimensional Mentalizing Questionnaire (MMQ): Translation, adaptation and psychometric properties in a Chilean population.”*

*This paper reports on the translation and adaptation of the Multidimensional Mentalizing Questionnaire (MMQ) into Spanish for a native Spanish-speaking sample in Chile. The study examines the psychometric properties and internal consistency of the translated MMQ. Convergent evidence for validity is reported by correlating MMQ subscales with the Interpersonal Reactivity Index (IRI) and Empathy Quotient (EQ). Divergent evidence for validity is assessed by correlating aggressive traits using the Buss-Perry Aggression Questionnaire (BPAQ). The study also explores gender and age differences. Results reveal positive correlations between good mentalizing and empathy, particularly cognitive empathy, supporting the significance of positive mentalization in empathy. Negative mentalization is associated with difficulties in perspective-taking and social skills, as well as aggressive traits. Gender differences in mentalizing capacities are observed, and negative aspects of mentalization decrease with age.*

*The investigators are to be commended for undertaking such an ambitious project. However, I do have several suggestions for improving the ms., addressed below.*

*First, the authors write of “convergent validity,” and divergent validity.” There is only one validity, but there can be various sources of evidence for validity, such as convergent evidence and divergent evidence.*

**Response 1:**

**We thank the reviewer’s comment. Following his suggestion, we modified the manuscript, referring to convergent and divergent evidence instead.**

*Second, the authors apparently conducted only one CFA, presumably testing a common factors model, whereas I recommend conducting an assessment of variance composition to determine if a total score can be modeled with alternative structures including bifactor, hierarchical, and unidimensional models. It appears that a common factors model was tested, in which responses to the MMQ were used as indicators of the hypothesized latent factors, with each item-level indicator loading on only one factor. The purpose of this model is to confirm the factor structure found in the EFA. Next, a bifactor model should be investigated. In the bifactor model, each item-level indicator is specified as having factor loadings on both a general factor (corresponding to the total scale score) and a group factor (corresponding to the subscale scores). All group factors should be specified as orthogonal to the general factor and to each other by fixing their intercorrelations to zero. A third alternative is the hierarchical model, in which the higher-order factor represents the general factor. The indicators of the higher-order factor are the lower-order latent variables corresponding to domain-specific constructs, in this case the subscales. The indicators for the lower-order latent constructs are the measured variables (e.g., item-level responses). Finally, a unidimensional model, in which all indicators load only on a general factor should be assessed.*

**Response 2:**

**Following the reviewer’s suggestion, to determine the best factor structure we tested 3 additional models and reported them in the manuscript: bifactor, one-factor, and hierarchical factor model. As can be seen in Table 3, our results show that common factors model had the best fit indexes compared to the 3 additional models.**

*Third, the authors compared different groups based on gender and age. Typically, with newly adapted scale one, to do this requires the assessment of whether or not the groups understand the scale in the same way. Whether or not particular scales are understood in the same way by men and women and younger and older people can be examined quantitatively through the assessment of measurement equivalence/invariance (ME/I) across age groups. This is a highly technical psychometric analysis that focusses on whether certain parameters resulting from confirmatory factor analyses are the same across the groups. When these parameters – factor loadings, item intercepts, and residuals – are the same for all groups, we can say that the different groups based on gender and age understand the scores of the scale in the same way, and we can reliably compare their men scores (Cheung and Lau, 2012; Kline, 2016). If they are not the same, then construct bias is likely present, and therefore we cannot have confidence in the results when comparing their scores on the scale. Assessing ME/I is clearly fundamental to good social science, but it is an oft-ignored prerequisite to comparing the scores of members of different groups on a scale, creating what a recent study has labeled “hidden invalidity” in the research literature (Hussey and Hughes, 2020).*

**Response 3:**

**A measurement invariance analysis was carried out using gender as group variable. We followed the recommendations of Wu and Estabrook (2016). These authors are concerned with how the threshold model is identified, and provide evidence to show that some constrains are needed to test the hypothesis that the instrument is measuring the same way different groups.**

**Ref: Wu, H. & Estabrook, R. (2016). Identification of Confirmatory Factor Analysis Models of Different Levels of Invariance for Ordered Categorical Outcomes. *Psychometrika*, 81(4), 1014–1045. doi:10.1007/s11336-016-9506-0**

**With respect to the age-based invariance analysis, our sample is predominantly young, so there is no clear balance with respect to the possible groups (see Figure below). If, for example, the median is used, one group would be homogeneous in terms of age, while another (the older group) would not. In addition, since we are not dealing with natural groups in our sample, we have no clear criteria to establish a cut-off point by age. We do acknowledge, however, that an analysis of invariance is important to draw conclusions regarding the correlation between the MMQ and age.**

*Furthermore, because measurement invariance testing is limited by yielding only a binary decision (invariance or non-invariance), we recommend performing a series of post-hoc bootstrap confidence interval tests, as recommended by Cheung & Lau (2012), to examine the statistical significance of any between-group difference on parameters (factor loadings and item intercepts) if the Δχ2 or ΔCFI did not support invariance. We also recommend calculating the effect size for measurement non-invariance using the DMACS, the SEM equivalent of a Cohen’s d (effect size for means and covariance structures (MACS; Nye & Drasgow, 2011; Nye et al., 2018). Based on simulation data, in which researchers manipulated the level of non-invariance in a model from small (i.e., not practically meaningful) to large (Nye et al., 2018), DMACS were interpreted using the following effect size cutoffs: .40 (small), .60 (medium), and .80 and greater (large).*

**Response 4:**

**Considering that our analysis of invariance showed no differences across gender, we did not perform these analyses.**

*Finally, the authors investigate evidence for validity using raw scores. Since raw scores contain error; therefore, I recommend comparing latent scores using structural regression (in Structural Equation Modeling).*

**Response 5:**

**In the revised manuscript, we report the correlations between the subscales of the MMQ with the latent scores and the raw scores. There were some changes in magnitude but not in direction. In the case of the correlations between the other instruments and the subscales of the MMQ, we report both the correlation between the latent scores of the MMQ and the raw scores of the other scales and between the raw scores of all scales and subscales.**

*References
Cheung, G. W., Lau, R. S. 2012. “A Direct Comparison Approach for Testing Measurement Invariance.” Organizational Research Methods 15:167-198.
Hussy, I., Hughes, S. (2020). Hidden Invalidity Among 15 Commonly Used Measures in Social and Personality Psychology. Advances in Methods and Practices of Psychological Science 3:166-184.
Kline, R. B. 2016. Principles and Practice of Structural Equation Modeling (Fourth Ed.). New York: Guilford.
Nye, C. D., Bradburn, J., Olenick, J., Bialko, C., Drasgow, F. 2018. “How Big Are My Effects? Examining the Magnitude of Effect Sizes in Studies of Measurement Equivalence.” Organizational Research Methods 22: 678-709.
Nye, C. D., Drasgow, F. 2011. “Effect Size Indices for Analyses of Measurement Equivalence: Understanding the Practical Importance of Differences Between Groups.” Journal of Applied Psychology 96:966–980.*
