# Supplementary material for: Spanish version of Multidimensional Mentalizing Questionnaire (MMQ): Translation, adaptation and psychometric properties in a Chilean population
Source: PLoS One. 2024 Mar 18;19(3):e0296691. doi: 10.1371/journal.pone.0296691 (PMC10947660; doi:10.1371/journal.pone.0296691)
Supplement: Attachment — Submitted filename: PONE review R1 11-06.docx [file pone.0296691.s002.docx]

Thank you for the invitation to review the first revision (R1) of the “Spanish version of Multidimensional Mentalizing Questionnaire (MMQ): Translation, adaptation and psychometric properties in a Chilean population.”

This paper reports on the translation and adaptation of the Multidimensional Mentalizing Questionnaire (MMQ) into Spanish for a native Spanish-speaking sample in Chile. The study examines the psychometric properties and internal consistency of the translated MMQ. Convergent evidence for validity is reported by correlating MMQ subscales with the Interpersonal Reactivity Index (IRI) and Empathy Quotient (EQ). Divergent evidence for validity is assessed by correlating aggressive traits using the Buss-Perry Aggression Questionnaire (BPAQ). The study also explores gender and age differences. Results reveal positive correlations between good mentalizing and empathy, particularly cognitive empathy, supporting the significance of positive mentalization in empathy. Negative mentalization is associated with difficulties in perspective-taking and social skills, as well as aggressive traits. Gender differences in mentalizing capacities are observed, and negative aspects of mentalization decrease with age.

The authors have responded to all of my suggestions.
