# Supplementary material for: Spanish version of Multidimensional Mentalizing Questionnaire (MMQ): Translation, adaptation and psychometric properties in a Chilean population
Source: PLoS One. 2024 Mar 18;19(3):e0296691. doi: 10.1371/journal.pone.0296691 (PMC10947660; doi:10.1371/journal.pone.0296691)
Supplement: Attachment — Submitted filename: ResponsetoReviewers_MinorChanges.docx [file pone.0296691.s003.docx]

Response to editor

We thank the editor for his comments and suggestions, which will surely contribute to the quality of our work. We have addressed each one in the text (in red font). We specify those additions in what follows, within quotes and in italics, linking them to each comment.

**1.- Summary and State of the Art**

**The summary provides a clear and concise overview of the purpose of the study, its methodology, and the main findings. The contextualisation of the MMQ and the rationale for its adaptation to Chilean Spanish are well supported. However, a more detailed discussion of how the MMQ compares and contrasts with similar instruments already existing in the Spanish-speaking context is missing, which would strengthen the rationale for this specific adaptation.**

ANSWER: We thank the editor for the comment. We have included in the abstract a reference to the fact that this would be the first scale adapted to the Chilean population that measures, through self-reporting, people's perception in their daily experiences with mentalization. We have further elaborated on this in the article, specifically following the editor’s comments 2, 3, and 5.

*“The availability of the Spanish translation of the MMQ, the first self-reporting scale measuring mentalization adapted to Chilean population, contributes to research aiming to understand its relationship with other psychological phenomena in different cultural context and facilitating clinical interventions with different population groups. We therefore encourage further investigation into cultural, gender and age differences in mentalization.”*

**2.- Problematisation and Research Question**

**The problematisation and formulation of the research question are adequate. The need for a validated instrument to measure mentalisation in the Chilean population is clearly identified. However, it would be beneficial to further explore how this study specifically contributes to clinical practice and mental health research in Chile.**

ANSWER: In the introduction, we have elaborated on the contributions of the MMQ in the clinical and mental health context.

*“Indeed, as mentioned above, mentalization is associated with mental health at the level of relational difficulties and interpersonal stress in schizophrenia and antisocial and borderline personality disorders. Moreover, suicidal behavior could also be linked to a low capacity for mentalization (44, 45). Thus, early detection of low levels of mentalization could serve both prevention and management in therapeutic interventions such as Mentalization-Based Therapy (46-48). In this regard, it has also been proposed that mentalization is one of the primary tools for establishing therapeutic settings that could lead to a good alliance between therapist and patient. Finally, the MMQ could also be applied to therapists in training, serving to identify shortcomings and recommend the development of mentalizing skills (47, 49, 50).”*

**3.- Hypotheses and Objectives**

**The objectives are clear and relevant, focusing on translating, adapting and evaluating the psychometric properties of the MMQ. However, a more specific hypothesis about the expectations of adaptation, especially in terms of cultural constructs or demographic differences, could have added depth to the study.**

ANSWER: As the study's focus was exclusively on examining the psychometric properties of the Chilean adaptation of the MMQ, we did not formulate specific objectives or hypotheses for cross-cultural comparisons. Additionally, exploratory analyses were conducted for gender and age comparisons. One limitation of our study is the age distribution within the sample, which precludes conclusive results due to a significant decrease in sample size as age progresses. We address these limitations in greater detail in the discussion, emphasizing their relevance to future research considerations.

*“In conclusion, the Spanish adaptation of the MMQ presented in this work demonstrates robust psychometric properties in the Chilean population. The study provides evidence of how the different dimensions of mentalizing may be related to empathic abilities and aggressive behaviors. Considering that the original version was developed in Italian, it is crucial to address potential cultural differences, as they may influence the behavioral expression of mentalization and item functioning (85, 86). Therefore, we advocate for cross-cultural studies that, among other questions, test cultural invariance. Additionally, exploring the relationship between age and mentalization and other pertinent demographic analyses in forthcoming investigations is crucial. Finally, the adaptation of the MMQ presented in this paper is the first tool that will serve for self-administered measurements of mentalization in Chile, providing a standardized and valid measure that can inform psychological treatment and research.”*

**4.- Methodology**

**The methodology is rigorous, with a detailed focus on translation, adaptation and analysis of the factor structure of the MMQ. The inclusion of measurement invariance analysis to examine gender differences is a strength. However, the predominantly young sample and the absence of an age-based invariance analysis limit the generalisability of the results. In addition, it would be useful to elaborate more on how item deletion decisions were handled and justified during adaptation. This is in order to safeguard the methodological and statistical process.**

ANSWER: We thank the editor for these observations. To address the first one, we added a caveat in the discussion as follows:

*“Moreover, future work should address the generalizability of our findings across life span through invariance analysis.”*

ANSWER: Also, we added the following text to address the second comment, further explaining the rationale for modifying the original scale by not including five items.

*“Aiming to improve the fit and, at the same time, safeguard the simplicity and the parsimony of the model, we look for items that correlated with one or more additional factors different than the one to which they were initially associated based on theoretical and empirical grounds. Specifically, we examined the modification indices provided by the Lavaan package v. 0.6.15 (61) to identify items to which adding an extra parameter, i.e., a factor weight, substantially improved the fit indicators of the model. Following this rationale, five items exhibited substantial cross-loadings (18, 26, 30, 31, and 32). Three of these items originally belonged to the Reflexivity subscale, which had the largest number of items, and the remaining two belonged to Ego Strength, the second largest subscale. As shown in Table 3, the model’s fit indicators improved substantially by removing those items, placing them within the accepted psychometric standards for the common factors model (62).”*

**5.- Discussion and Conclusions
The discussion is comprehensive in terms of the interpretation of the results, highlighting the relationships between mentalisation, empathy and aggressive traits. Consideration of limitations and suggestions for future research is adequate. However, it would be valuable to include a more detailed discussion of the practical implications of these findings for mental health professionals in Chile.**

ANSWER: In the discussion, we have elaborated the practical implications of the MMQ results in the clinical and mental health context.

*“In conclusion, the Spanish adaptation of the MMQ presented in this work demonstrates robust psychometric properties in the Chilean population. The study provides evidence of how the different dimensions of mentalizing may be related to empathic abilities and aggressive behaviors. Considering that the original version was developed in Italian, it is crucial to address potential cultural differences, as they may influence the behavioral expression of mentalization and item functioning (85, 86). Therefore, we advocate for cross-cultural studies that, among other questions, test cultural invariance. Additionally, exploring the relationship between age and mentalization and other pertinent demographic analyses in forthcoming investigations is crucial. Finally, the adaptation of the MMQ presented in this paper is the first tool that will serve for self-administered measurements of mentalization in Chile, providing a standardized and valid measure that can inform psychological treatment and research.”*

**6.- Concluding comment
The article is valuable and contributes significantly to the psychometric literature, especially in the context of mental health in Chile. The corrections made by the authors have substantially improved the manuscript, so these minor corrections, particularly with regard to the discussion of the specific cultural context and the generalisation of the results, will allow for an adequate writing***.*

We thank the editor for this comment and totally agree with it. We hope we have addressed all issues raised.
